# Online coding of the Brief Observation of Social Communication Change (BOSCC) to capture treatment response in minimally verbal children with autism spectrum disorder

**DOI:** 10.1177/20552076251347105

**Published:** 2025-06-17

**Authors:** Christina Toolan, Hannah Singer, Stephanny Freeman, Tanya Paparella, Rebecca Elias, Maksim Tsvetovat, Catherine Lord

**Affiliations:** 1 Department of Child Development, California State University, Carson, CA, USA; 2 Department of Psychiatry, University of California, Los Angeles, CA, USA; 3 Department of Education and Information Studies, University of California, Los Angeles, CA, USA; 4 Department of Psychiatry, Children's Hospital Los Angeles, Los Angeles, CA, USA; 5 Department of Psychiatry and Behavioral Sciences, Keck School of Medicine, 5116 University of Southern California, Los Angeles, CA, USA; 6 Open Health Network, Mountain View, CA, USA

**Keywords:** Autism spectrum disorder, children, coding behaviors, digital, observational studies

## Abstract

The Brief Observation of Social Communication Change (BOSCC) measures subtle changes in social communication behaviors in children with autism spectrum disorder (ASD). In this brief communication, we examine an online platform that was developed to streamline BOSCC coding and support the development of machine learning-based automatic coding. This study found online coding was comparable to manual coding, capturing social communication changes among children receiving ASD intervention. This advancement offers an efficient alternative to paper-and-pencil coding methods, increasing access and usability of this innovative measure.

## Introduction

Difficulty with social communication (SC) is a hallmark of autism spectrum disorder (ASD) and is a critical target of many ASD interventions. The Brief Observation of Social Communication Change (BOSCC) is a novel observational assessment that provides a standardized and efficient method of measuring changes in SC behaviors in autistic children over multiple timepoints.^1^ The BOSCC addresses several limitations of treatment response measures used in autism intervention studies, including potential bias in parent- and clinician-reported outcomes and capturing the heterogeneity and subtlety in behavioral change over a short period of time.^2^ Previous studies have demonstrated that the BOSCC detects changes in SC behaviors of minimally verbal children and adults with ASD using a manual coding procedure.^1,3–7^ The BOSCC has also been tested across a range of early autism intervention models for young children^8^ and across home and school contexts.^9^ The BOSCC coding scheme has also been applied to videotaped segments of the Autism Diagnostic Observation Schedule (ADOS)^10^ conducted with young minimally verbal children, with the BOSCC and BOSCC-coded ADOS showing comparable results.^11,12^ Another version of the BOSCC has also been used to detect changes in SC behaviors in young children with phrase speech.^13^

The aforementioned studies on the BOSCC have used manual coding to support their results. Manual coding of the BOSCC is a paper-and-pencil process, which involves watching videos on a screen, writing notes on paper by hand, referencing of the coding scheme, and manual entry of codes into a database. While effective, manual coding is time-consuming, can be inefficient, and is prone to human error. As highlighted in commentaries on the BOSCC,^14,15^ digitizing the coding process and automating certain coding decisions (e.g. timestamps) would greatly benefit clinicians and researchers using the BOSCC by providing a more efficient and reliable method for measuring individuals’ SC changes, ultimately making the BOSCC more widely useable.

To address this need, an online coding platform was developed to streamline the BOSCC coding process. This platform also represents a step toward the development of automatic coding using machine learning of audio-visual information for children with ASD. While similar efforts have been made in autism diagnostic classification decisions using machine learning with adults and children^16–18^; few have examined treatment response measures or change over time in young children. These efforts build on previous work involving automatically derived measures of child language,^19^ automated activity recognition and abnormal behavior recognition from the ADOS,^20^ and automated treatment response measures in children with ASD using BOSCC data.^21^

In this brief communication, we examine a small-scale study using secondary data analysis. We aimed to (1) determine if online-coded BOSCC data, like manually coded data, captures changes in SC behaviors over the course of treatment, (2) examine inter-rater reliability of BOSCC online coding, and (3) compare item-level and summary codes across manual and online coding methods.

## Method

This study is a secondary analysis of a subset of data collected for a larger assessment study (IRB#19-000220). The study took place in a large, urban academic medical center on the West Coast. Data for the present study was collected between October 2018 and March 2020.

The sample for the current study included 84 observations from 31 unique participants (*n*_female_ = 7, *M*_age_ = 46.39 months, *SD* = 13.89, range = 29–80 months). Inclusion criteria for the study were (1) an autism diagnosis, (2) minimal or limited use of speech (not using phrases), (3) the ability to walk without assistance, and (4) normal or corrected hearing and vision. All participants were children who were enrolled in a short-term, center-based, comprehensive, intensive partial hospitalization program for young children with ASD. Parents provided their written consent for participation in the study. Exclusion criteria included those who exhibited additional impairments (i.e. blindness and deafness) or a diagnosis of cerebral palsy.

The BOSCC is a 12–14 minute videotaped play interaction between a child and adult play partner blind to treatment status.^1^ In this study, the version of the BOSCC designed for minimally verbal children (BOSCC-MV) was used. The BOSCC-MV includes a standardized set of toys intended for joint use by the child and adult play partner. Play partners are provided with minimal instructions, as the assessment is intended to capture SC behaviors in naturalistic play interactions. The BOSCC was completed at entry, midpoint (5 weeks of intervention), and exit (10 weeks of intervention) with undergraduate research assistants serving as the play partner.

BOSCC coding consists of 15 items coded on a 6-point scale ranging from 0 (abnormality is not present) to 5 (abnormality is present and significantly impairs functioning). Decision trees are used to guide the coding process. Items are categorized into two domains: SC (SC; eight items) and restricted and repetitive behaviors (RRBs; four items); these 12 items are summed to yield a Core Total score (see Grzadzinski et al.^1^ for a full description of BOSCC items).

An additional three items (anxiety level, disruptive behavior/irritability, and anxious behaviors) were coded to indicate the presence and severity of other abnormal behaviors commonly seen in children with ASD. These items can be used to determine the validity of a BOSCC—that is, if the BOSCC observation is representative of the child's typical behaviors.^1^ These behaviors rarely received scores other than 0 in this sample. The items were excluded from the present analyses due to little variability, as has been done in previous studies.^1,8^

BOSCC videos were scored by research-reliable coders (intraclass correlation (ICC) > 80% in training) who were blind to timepoint. Study videos were then randomly assigned to coders. Each BOSCC video was coded twice: once manually and once using the online coding platform. Manual coding involved watching the video on screen, hand-writing notes and timestamps on paper, and referencing the coding scheme to score. The online coding platform utilized embedded video playback, with notes typed and automatically timestamped, hotkeys enabled as shortcuts to commonly used codes, and coding decision trees incorporated into the virtual system. The same coder coding the same video both manually and online was minimized (*n* = 10, 16% of videos). Approximately 18% of videos across each platform were coded by multiple coders for inter-rater reliability.

### BOSCC online coding platform

The BOSCC online platform was designed and developed by a collaborative team of researchers and engineers. The online platform served two primary functions mirroring the manual coding process: (1) annotating the video with coding notes, and (2) guiding the coding process (i.e. assigning scores to each BOSCC item) using decision trees. There were key features that made the online process unique from manual coding. For video annotation on the online platform, the video and coding notes were displayed on screen at the same time, which allowed the coder's attention to remain on the screen during the entire annotation process. This was in contrast to manual coding, which involved watching the video on a screen and writing notes on paper—requiring coders to shift attention back and forth from screen to paper. Online coding allowed coders to include their notes with automatic timestamps as the video played, with a running timeline of notes displayed. Hotkeys for various commonly used codes were included to make notetaking more efficient.

After the video segments played and were annotated, the user would be led to the coding decision trees. For each item of the BOSCC, notes from the video annotation would be displayed on the screen to help guide coders through each decision. Coders could filter their notes by relevant hotkeys pertaining to each item. In contrast, manual coding required coders to simultaneously reference a copy of the coding scheme and their handwritten notes to guide their coding decisions. The online platform also required coders to provide a response to each question in the decision tree as a way to reduce human error.

In addition to facilitating the coding process, the online platform was also used as a repository for BOSCC videos and data management. Users could use the platform to delegate coding assignments (videos) to different coders, review coding results, and track inter-rater reliability.

### Statistical analysis

To examine change over time by coding method (Aim 1), we separately analyzed manual and online coded BOSCC data over time (entry to exit). Wilcoxon signed-rank tests were used to account for non-parametric data. For each method, we examined changes in BOSCC SC, BOSCC RRB, and Core Total scores. Results of manual and online coded BOSCCs change over time were compared. Coding inter-rater reliability (Aim 2) was examined using ICCs. Both manual and online coding methods were examined, with separate ICCs run for BOSCC SC, BOSCC RRB, and Core Total scores for each. To compare item-level and summary codes across coding methods (Aim 3), we used independent *t*-tests to compare item-level and summary-level means between manual and online coding scores.

## Results

When examining changes in BOSCC by coding method (Aim 1), there were significant decreases (indicating improvement) in BOSCC SC scores from entry to exit using both manual (Wilcoxon signed-rank *Z* = –2.34, *p* = 0.019, ES = −0.33) and online coding (Wilcoxon signed-rank *Z* = –3.07, *p* = 0.002, ES = −0.43). There were no differences in BOSCC RRBs across time with either coding method. There were no differences in Core Total scores from entry to exit using manual coding, but there was an improvement in Core Total scores when using online coding (Wilcoxon signed rank *Z* = –1.96, *p* = 0.050, ES = 0.28).

Inter-rater reliability was high across manual (ICC_SC_ = 0.975, ICC_RRB_ = 0.982, ICC_CoreTotal_ = 0.985) and online coding (ICC_SC_ = 0.957, ICC_RRB_ = 0.892, ICC_CoreTotal_ = 0.963) methods (Aim 2).

**Figure 1.**
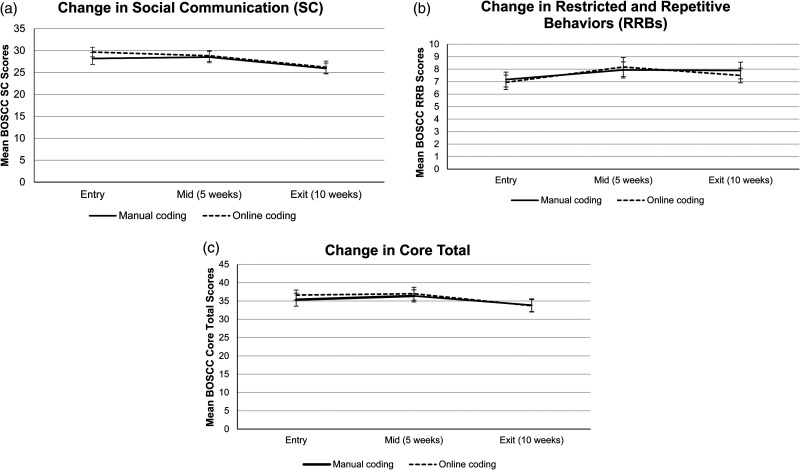
(a) Change in BOSCC SC scores over time across manual and online coding methods. (b) Change in BOSCC RRBs scores over time across manual and online coding methods. (c) Change in BOSCC core total scores over time across manual and online coding methods. These line graphs depict changes in average BOSCC: (a) SC, (b) RRBs, and (c) Core Total scores at entry, midpoint (5 weeks), and exit (10 weeks) across manual and online coding methods. There were significant decreases in BOSCC SC or BOSCC RRB scores from entry to exit using both coding methods. There were no significant differences in BOSCC Core Total scores from entry to exit using manual coding, but there were significant differences when using online coding. BOSCC SC, RRB, and Core Total scores were comparable across coding methods. Error bars represent the standard errors of the mean.

BOSCC: Brief Observation of Social Communication Change; SC: social communication; RRBs: restricted and repetitive behaviors.

There were no significant differences in BOSCC summary scores across manual and online coding methods (SC: *t*(165)=−0.70, *p* = 0.485; RRB: *t*(165) = .23, *p* = 0.820; Core Total: *t*(165)=−0.45, *p* = 0.654; Aim 3) (see Figure 1(a) to (c)). There were also no significant differences in item codes across the manual and online coding methods (see Table 1).

**Table 1.** Comparison of Brief Observation of Social Communication Change (BOSCC) item codes across manual and online coding methods.

	Manual (*M*)	Online (*M*)	*t*	*p*
Eye contact	3.69	3.46	1.94	0.054
Facial expressions	3.71	3.79	−0.67	0.503
Gestures	3.90	3.99	−0.47	0.637
Vocalizations	2.73	2.80	−0.30	0.762
Integration	3.96	4.07	−0.68	0.497
Social overtures	3.49	3.71	−1.21	0.228
Social responses	2.91	3.15	−1.27	0.206
Engagement	3.22	3.37	−1.02	0.309
Play	3.17	3.23	−0.53	0.595
Sensory interests	1.26	1.19	0.33	0.741
Mannerisms	1.81	1.90	−0.35	0.726
Restricted, repetitive behaviors	1.40	1.20	1.08	0.281

## Discussion

Results of the study indicate that online BOSCC coding, like manual BOSCC coding, can measure subtle changes in SC over time. In the current sample, SC improvements were noted in young minimally verbal children with ASD across manual and online coding methods, and the results from both were very similar. Further, online BOSCC coding was comparable to manual coding in the RRB domain. Changes in RRBs were detected, though improvements were not identified for these subjects over time with either coding method. Core Totals were also comparable across both coding methods, though the online coding procedure uniquely detected a small yet significant improvement. More work is needed to determine if this pattern is replicated across samples.

There was excellent inter-rater reliability with online coding, as with manual coding. Manual and online BOSCC coding produced comparable item codes and summary scores. Results are proof of concept that the BOSCC can be coded using an online platform, with similar results to manual coding. This is advantageous as online coding has the potential to reduce the coder burden.

Moving BOSCC coding online increases the accessibility of the BOSCC to clinical researchers. It also reflects a growing shift toward telehealth, necessitated by the COVID-19 pandemic.^22,23^ Though our data were collected prior to COVID-19 restrictions—and though those restrictions have since been lifted—there is continued interest in using flexible and remote applications of autism assessments, services, and research. Online applications of the BOSCC could be used to facilitate more consistent and timely evaluations of treatment responses in autistic populations and reach a wide range of research and clinical practices.

There are limitations of this study that warrant further investigation. First, this was a preliminary study with a small sample size, which limits the generalizability of our findings. Additionally, we only examined online coding of the BOSCC MV, and only with young children enrolled in an intensive early intervention program. Future work could examine other versions of the BOSCC (e.g. phrase speech or verbally fluent) with participants across a wider age range and across various contexts. In the future, we would also like to examine the potential additional benefits of online coding from a user and evaluation perspective, comparing metrics such as time spent coding or errors on training videos. A technical limitation at the time that the online coding platform was developed was the quality of neural networks we could employ for auto-coding the videos; multimodal LLMs now have the ability to be trained to automatically detect relevant expressions and code videos with minimal supervision. We plan to continue to develop alternative digital approaches to analyzing clinical observations of children with autism.^24^

## Conclusions

This study examined the use of an online coding platform to code the BOSCC, digitizing the time-consuming pencil-and-paper manual coding process and representing a step in developing automatic coding of audio-visual information for children with ASD. Coding the BOSCC using an online platform produced comparable results to coding the BOSCC manually. Both were able to capture subtle changes in SC in young minimally verbal children with autism.

Using an online platform BOSCC coding is exciting as it supports a streamlined coding process, allowing for efficient coding and data management. This advancement also paves the way for the automatic coding of audio-visual information to assess treatment response in children with ASD. Transitioning to online BOSCC coding—as this study proves is feasible—is not just a technical upgrade, it represents a leap toward making this valuable assessment tool more accessible and efficient. Finally, online coding of the BOSCC represents a move toward greater accessibility of the BOSCC to a wider audience of clinicians and researchers. Such an advancement could ultimately improve the quality of care and support provided to children with ASD.
